# Dr. Friedmann’s Curative and Prophylactic Vaccination against Human Tuberculosis

**Published:** 1913-04

**Authors:** 

**Affiliations:** Kissingen, Germany


### BUFFALO MEDICAL JOURNAL

VOLUME 68 APRIL, 1913 No. 9

### ORIGINAL ARTICLES

### Dr. Friedmann's Curative and Prophylactic Vaccination Against Human Tuberculosis.

Short Report by DR. HESSE
Kissingen, Germany

AT the London Medical Congress in 1900 Robert Koch announced that the tubercle bacilli of man were not pathogenic for cattle and a little later Behring proved that cattle that were vaccinated with human tubercle bacilli were considerably more resistent against bovine tuberculosis. Immunization against tuberculosis which had never been achieved by tuberculin was accomplished by the inoculation of living tubercle bacilli taken from an animal of a different species. These experiments were continued and confirmed by others and of these F. Klemperer came to the following conclusion: Tuberculosis is curable if treated by immunization with living tubercle bacilli that are not injurious to the animal in question. As Koch himself considered this method promising, work was continued on that principle. Some years ago Friedmann announced that he had succeeded in immunizating guinea pigs with the tubercle bacilli of tortoises.

Vaccination, with living bacilli of a different species, however, nearly always causes abscesses at the vaccination spot and for that reason all attempts on human beings were impossible.

In November, 1912, Friedmann read a paper before the Berlin Medical Society announcing that after many years of hard work he had succeeded in getting a breed of Tbc. from tortoises which showed a higher degree of harmlessness (avirulence) than all predecessors. The result of experiments performed on guinea pigs with this breed of Tbc. was that the prevaccinated animals, though not immune, lived considerably longer after subsequent artificial tuberculous infection than animals that had not been prevaccinated (confirmed by Prof. Orth). Besides, that breed of Tbc. was absolutely harmless to guinea pigs even in large doses and hardly produced any infiltrations and so Friedmann no longer hesitated to commence experimenting on man. Also in these experiments on human beings the remedy proved to be harmless even in large doses, no matter in what way it was given, under the skin, into the muscles, into the veins, per os, into the eyes or directly on open tuberculous wounds. Another important observation made by Friedmann, was that intravenous application, though it brings on almost immediate signs of healing, very soon loses its effect, and the healing process comes to a standstill. Friedmann found that the most effective way of applying the remedy is injection into the muscles, once, twice or three times, even oftener at long intervals. It is necessary that at the place of vaccination a swelling should form from the size of a nut to that of a small apple, and this swelling ought to be resorbed slowly. As long as this swelling exists and during its slow resorption the healing process proceeds. Should the resorption prove incomplete or should, for reasons still unknown, the infiltration get soft so that an abcess or discharge sets in, the healing process often stops, and further injections would prove useless and would only cause other abcesses or filtrations.

Friedmann believes he has discovered a way of preventing the tendency to abcess formation. He recommends injecting the first dose into the veins and then the subsequent ones into the muscles, and thinks that the intravenous dose increases the resorption capacity of the body. He calls this method the "Simultanmethode."

Up to the time of his lecture, Friedmann had treated about 1200 persons with tuberculosis of all kinds, and he gave an account of his amazing successes. Very advanced cases of tuberculosis of the bones, joints and glands were treated with the new remedy and were cured in a comparatively short time, while under the control of other doctors. Tuberculosis of the lungs is said to make rapid improvement in all cases except those where the breaking down is too far advanced. Tuberculosis of the kidneys and the bladder as well as that of the testicles were treated successfully; the same was said of tuberculosis of the skin, of the larynx and of the so-called scrofulous diseases of children.

Simultaneously with these cures, prophylactic injections were performed on children that were in any way threatened by the disease. Up to the date of his lecture Friedmann had inoculated 335 children, aged from one hour to three years. The injections, all of which had been made into the muscles, occurred without accident and so far have done no damage. On the contrary, the vaccinated children are said to have developed in a remarkably healthy way.

A most animated and lengthy discussion followed the lecture and the demonstration of a certain number of healed and improved cases. At first several doctors spoke whose patients had been under Friedmann's treatment, and who had watched the results with him. As a matter of fact, the accounts of these gentlemen—I mention Erich Müller, Karfunkel, Konrad Küster, Hey-were all extremely favorable, and even discounting the natural enthusiam with which every new remedy is received, there still remains enough to cause amazement. Those who till then had not had the opportunity of testing Friedmann's method acknowledged that his ideas were theoretically well founded, and followed those lines that seem most promising. Thus Citron remarked, that of late years it has been more and more positively shown, that it is useless to try to cure or immunize with any dead virus. Every great success that has been achieved in this part of medical science, like Jenner's vaccination against smallpox, or Pasteur's vaccination against rabies, has been done with living virus, and therefore one may conclude that the same will be the case for tuberculosis. However, Citron continues, as long as we do not know the details of Friedmann's method, we must approach its practical application with great scepticism and care, for experience teaches us that a virus which seems to be avirulent may regain its virulent qualities under the changed circumstances of a different soil. Above all, the prophylactic vaccination can not yet be answered for—an opinion that was likewise expressed by other debators. It may be remembered as was mentioned by several authorities on this subject (Kausch, Aronson, Fritz Meyer Katzenstein) that with different kinds of tuberculine, as, for instance, that of Roesenbach or that of Fritz Meyer, good results have been achieved which are in no way inferior to Friedmann's. Therefore there is no reason to forsake the old methods as long as the new one is not more generally tried and known. Naturally, to be just, we must wait for the detailed publication which Friedmann promises to give regarding the preparation and the nature of his remedy. How often has our rash enthusiasm been disappointed. We must not forget that there is, perhaps, no other illness in which it is more difficult to form a correct opinion as to the effect of our numerous therapeutic measures. The individual cases of this illness are so different and changeable in their course that it is almost impossible to form a true prognosis, and many a case, especially of surgical tuberculosis, undergoes spontaneous cures.

The standpoint that the majority of German doctors take for the present on the subject of Friedmann's discovery has been well expressed in the declaration, which Prof. Bier made the other day in the Berlin Medical Society. He said: "It is certainly regretable that a preparation that has not been sufficiently tested should be praised in the foreign daily press as a great discovery and prominent remedy. Till now it has in no way stood its proof, especially as it is only in the hands of a single doctor and no sufficient control has yet been possible. In case as has happened before, the remedy should not prove satisfactory, it would do great damage to the reputation of German Medical Science and the Medical Profession, if the foreign press published without any contradiction that in the principal German Medical Society a favorable opinion had been given by well known Berlin doctors, for it apparently was not mentioned in the foreign press that some of the most prominent doctors had expressed grave doubts on Friedmann's remedy. "I must therefore publicly protest that my name should not be misused as recommending a remedy of the effectiveness of which I am yet in no way convinced. This declaration, of course, is not an opinion on the value of the remedy."

---

DERMOTROPISM.—The cutaneous eruptions caused by certain drugs form a subject for an interesting article by Bernard Fantus and A. W. Stillians, September *American Journal of Dermatology*). They conclude that dermotropism is, in general, in proportion to systemtic irritant action; that non-irritant substances are not dermotropic unless they produce hyperemia or long-continued ischemia of the skin, or unless they give rise to anaphylactic reactions; and that anaphylaxis may play an important role in determining dermotropic reactions to drugs. The most common drug eruptions are the following:

Bromides and iodides—Papulo-pustules.
Arsenic—Erythema, vesicles, urticaria and papulo-pustules.
Quinin—Erythema, urticaria, petechiae and vesicles.
Antipyrin—Erythema, papulo-pusules and urticaria.
Belladonna, stramonium, hyoscyamus—Erythema.
Opium and morphin—Erythema and urticaria.
Chloral—Erythema.
Horse serum or diphtheria antitoxin—Erythema and urticaria.

---

RECTAL PROLAPSE IN CHILDREN.—Camillo Vidakovich, Klausenburg, Hungary (*Zent. fur Chir.,* October 12, 1912). For acute rectal prolapse in children the author, after replacing the gut, applies a broad pelvic belt of adhesive plaster placed low enough to hold the buttocks together without blocking defecation. The belt is reinforced by two fairly broad strips which cross in the midline at the lower edge of the belt, posteriorly, and are brought around either thigh, ending between the upper and middle thirds in front. The splint is removed as often as soiled, and kept for one month.

